# Development of Automated Patch Clamp Technique to Investigate CFTR Chloride Channel Function

**DOI:** 10.3389/fphar.2017.00195

**Published:** 2017-04-07

**Authors:** Arnaud Billet, Lionel Froux, John W. Hanrahan, Frederic Becq

**Affiliations:** ^1^Laboratoire Signalisation et Transports Ioniques Membranaires, Université de Poitiers – ERL7368, Centre National de la Recherche ScientifiquePoitiers, France; ^2^Department of Physiology, McGill University, MontrealQC, Canada; ^3^McGill Cystic Fibrosis Translational Research Centre, MontrealQC, Canada; ^4^The Research Institute of the McGill University Health Centre, MontrealQC, Canada

**Keywords:** CFTR, automated patch clamp, medium throughput assay, physiological temperature recording, current stability

## Abstract

The chloride (Cl^-^) channel cystic fibrosis transmembrane conductance regulator (CFTR) is defective in cystic fibrosis (CF), and mutation of its encoding gene leads to various defects such as retention of the misfolded protein in the endoplasmic reticulum, reduced stability at the plasma membrane, abnormal channel gating with low open probability, and thermal instability, which leads to inactivation of the channel at physiological temperature. Pharmacotherapy is one major therapeutic approach in the CF field and needs sensible and fast tools to identify promising compounds. The high throughput screening assays available are often fast and sensible techniques but with lack of specificity. Few works used automated patch clamp (APC) for CFTR recording, and none have compared conventional and planar techniques and demonstrated their capabilities for different types of experiments. In this study, we evaluated the use of planar parallel APC technique for pharmacological search of CFTR-trafficking correctors and CFTR function modulators. Using optimized conditions, we recorded both wt- and corrected F508del-CFTR Cl^-^ currents with automated whole-cell patch clamp and compared the data to results obtained with conventional manual whole-cell patch clamp. We found no significant difference in patch clamp parameters such as cell capacitance and series resistance between automated and manual patch clamp. Also, the results showed good similarities of CFTR currents recording between the two methods. We showed that similar stimulation protocols could be used in both manual and automatic techniques allowing precise control of temperature, classic *I*/*V* relationship, and monitoring of current stability in time. In conclusion, parallel patch-clamp recording allows rapid and efficient investigation of CFTR currents with a variety of tests available and could be considered as new tool for medium throughput screening in CF pharmacotherapy.

## Introduction

The cystic fibrosis transmembrane conductance regulator (CFTR) is a cAMP-dependent chloride (Cl^-^) channel whose encoding gene is mutated in the common autosomal recessive disease cystic fibrosis (CF; Riordan et al., 1989). Mutations of the *CFTR* gene have been grouped into six classes according to their effects on synthesis, maturation, and function of the CFTR protein. The most common CFTR mutation is the deletion of the phenylalanine 508 residue (F508del-CFTR), a mutation present on at least one allele in ∼90% of patients with CF. The F508del mutation induces misfolding of the nascent protein and retention in the endoplasmic reticulum (Cheng et al., 1990), reduced stability at the plasma membrane (Lukacs et al., 1993), abnormal channel gating with low open probability (Dalemans et al., 1991), and thermal instability, which leads to inactivation of the channel at physiological temperature (Wang et al., 2011; Liu et al., 2012).

Cystic fibrosis transmembrane conductance regulator pharmacotherapy is one major therapeutic strategy that involves identifying compounds that correct abnormal CFTR protein. Most research effort is directed at finding small molecules which interact directly with mutated CFTR to correct its folding and gating, and thus improve protein trafficking and channel function. Two compounds that have yielded promising results are the potentiator Ivacaftor, also called VX770 (Van Goor et al., 2009), which was approved by the Food and Drug Administration (FDA) in 2012 as Kalydeco^TM^ for patients over 12 years of age carrying the G551D mutation (5% of patients), and the corrector Lumacaftor, also called VX809 (Van Goor et al., 2011). Lumacaftor was approved for clinical use in combination with Ivacaftor for the treatment of F508del homozygous patients; however, this combination therapy, called Orkambi^TM^, provided only modest benefit (Boyle et al., 2014) indicating that more efficacious correctors are still needed. Therefore, the search for innovative small molecules and understanding their mechanisms of action remain a priority for CF therapeutic research. To this end, robust screening assays are essential for rapidly testing large numbers of compounds and performing informative concentration–response curves *in vitro*. As multiple CFTR defects need to be corrected, cell-based assays should monitor improvements in the trafficking, membrane stability, and channel function of rescued mutant CFTR.

One of the most powerful techniques for characterizing ion channels and studying their pharmacology is patch clamp recording. It allows the recording of channel currents in real time, in a biological environment, and with high resolution (Hamill et al., 1981). Unlike other functional assays such as Ussing chamber short-circuit current measurements, flux studies (using either radioisotopes or ion-selective electrodes), or fluorescence assays [membrane potential detection (Van Goor et al., 2006; Maitra et al., 2013) or yellow fluorescent protein (YFP) quenching (Galietta et al., 2001; Pedemonte et al., 2011)], patch clamping provides precise and complete electrophysiological information on ion channel function. Although conventional manual patch clamp (MPC) techniques have low throughput and are therefore not suitable for screening phase, automated patch clamp (APC) techniques are available and can be applied to investigate CFTR pharmacology.

Parallel planar APC provides a “medium throughput screening” technique with higher resolution than other screening methods and provides quantitative information on the electrophysiological properties of ion channels more rapidly than MPC (Farre et al., 2009). APC has become the gold standard for early safety assessment when examining cardiac hERG channel to new drug candidates (Polonchuk, 2012) but has not yet been widely used for CFTR pharmacology studies (Liu et al., 2009; Jacobsen and Sørensen, 2016; Brüggemann et al., 2017).

The objective of this study is to describe the application of APC technology to CFTR pharmacology. We recorded wt- and F508del-CFTR currents using planar APC under various conditions and compared the results with those obtained with MPC method.

## Materials and Methods

### Cell Culture

Baby Hamster Kidney (BHK) cells were cultured in DMEM/F12 medium supplemented with 5% FBS and 1% Penicillin/Streptomycin. To study CFTR current, BHK were stably transfected with pNUT-wt-CFTR or pNUT-F508del-CFTR plasmids using JetPeI reagent (Polyplus, Illkirch, France) according to the manufacturer’s instructions. Clones were selected by addition of methotrexate (500 μM) and CFTR expression validated by immunoblotting.

Chinese hamster ovary (CHO), HeLa, and CFBE41o [Cystic Fibrosis Bronchial Epithelial (CFBE)] cells were cultured as previously described (Kunzelmann et al., 1993; Grygorczyk et al., 1996; Hinzpeter et al., 2010). The CFBE cells developed in the laboratory of D. Gruenert had been transduced with wt- or F508del-CFTR containing the M470 polymorphism by Transzyme (Birmingham, AL, USA) and were kindly provided JP Clancy (Cincinnati Children’s Hospital Medical Center).

Cells were cultured at 37°C in 5% CO_2_ and were plated in 8.8 cm^2^ dishes for MPC and 21.6 cm^2^ dishes for APC. Patch-clamp experiments were performed 2–3 days after plating. APC needs cells in suspension, therefore, cells at 70–80% confluence were detached by incubation with Accutase (EMD Millipore, Billerica, MA, USA) for 5 min at 37°C, suspended in complete culture, centrifuged for 2 min at 1000 rpm, and resuspended in DMEM/F12 without serum. Prior to use, the suspension was placed at 37°C for 15–20 min to allow cells to recover from the detachment process.

### Patch Clamp Experiments

For conventional MPC, Cl^-^ currents were measured in the whole-cell patch-clamp configuration after compensating electronically for pipette capacitance in the cell-attached mode. Voltage-clamp signals were recorded using an Axopatch 200B amplifier connected to an analog/digital interface, Digidata 1440A, and were analyzed using pCLAMP 9 software (all from Axon Instruments Inc., Burlingame, CA, USA). Pipettes were pulled from borosilicate glass capillaries (GC150-TF10; Clark Electromedical Inc., Reading, UK) and connected to the head stage of the patch clamp amplifier through an Ag–AgCl wire. The resistance of pipettes in the bath solution ranged between 4 and 6 MΩ.

Automated whole-cell patch clamp recordings were obtained using two different systems. Most experiments were performed on the eight-channel Patchliner^®^NPC-16 workstation (Nanion Technologies GmbH, Munich, Germany), which was coupled to two QuadroEPC-10 amplifiers (HEKA Elektronik GmbH, Germany). APC procedures followed Nanion’s standard procedures and used Nanion’s high-resistance chips. Some experiments were also performed using the Qpatch^®^ 16X system (Sophion Bioscience, Ballerup, Denmark) with single-hole Qplates. Both chip models exhibited a resistance of 3–3.5 MΩ.

**FIGURE 1:**
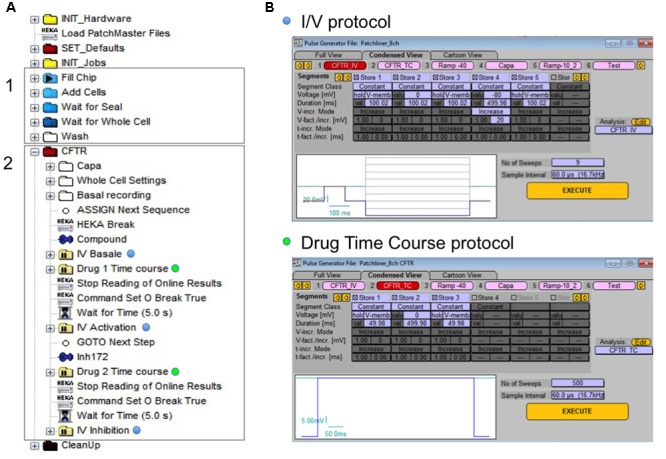
**Experimental procedure for automated patch clamp (APC) cystic fibrosis transmembrane conductance regulator (CFTR) recording. (A)** Tree of events for whole-cell configuration establishing (gray rectangle 1) and personalized CFTR recording (gray rectangle 2). **(B)** The two different protocols applied for CFTR recording: *I/V* protocol (blue dots) for current/voltage relationship visualization and drug time course (green dots) protocol to monitor the effect of compound in time.

Similar experiments were performed using APC and MPC. The holding potential was maintained at -40 mV throughout the experiment, and two voltage-clamp protocols were used to measure whole-cell CFTR currents. **Figure 1** shows the sequence of events (called *tree*) used on the Nanion software (**Figure 1A**) and protocols applied for CFTR recording (**Figure 1B**). The *tree* of events shows all steps leading to the whole-cell configuration (gray rectangle 1) followed by the CFTR recording parameters (gray rectangle 2). The current–voltage (*I*–*V*) relationship (*I*/*V* protocol, blue dots) was determined by pulsing from the holding potential of -40 mV to test potentials between -80 and +80 mV increasing in 20 mV increments. To monitor the current evolution under drugs application and confirm the absence of significant leak current; a single depolarization from -40 to 0 mV was applied every 5 s for 6–7 min (drug time course protocol, green dots).

### Medium and Chemicals

Similar external and internal solutions were used for MPC and APC. The external bath solution contained (in mM): 145 NaCl, 4 CsCl, 1 CaCl_2_, 1 MgCl_2_, 10 glucose, and 10 TES titrated with NaOH to pH 7.4. The osmolarity of the bath solution was 315 ± 5 mOsmol. The intrapipette solution contained (in mM): 113 L-aspartic acid, 113 CsOH, 27 CsCl, 1 NaCl, 1 MgCl_2_, 1 ethylene glycol tetraacetic acid (EGTA), 10 TES, and 3 MgATP titrated to pH 7.2 with CsOH. Osmolarity of the pipette solution was 285 ± 5 mOsmol. To facilitate giga-ohm (GΩ) seal obtaining with the Patchliner^®^system, the external solution was transiently replaced by a seal enhancer solution during the sealing procedures. The seal enhancer solution contained (in mM): 80 NaCl, 3 KCl, 10 MgCl_2_, 35 CaCl_2_, and 10 Hepes (Na^+^ salt) titrated with HCl to pH 7.4. Osmolarity of the seal enhancer solution was 298 ± 5 mOsmol. After stable sealing of the cell, the seal enhancer solution was replaced by the external solution before performing the whole-cell configuration and recordings. Recordings were performed at room temperature (20–25°C) unless stated otherwise.

VX809 and VX770 were from Selleckchem (Houston, TX, USA) and CFTR_inh_-172 was from Calbiochem (San Diego, CA, USA). All other products were from Sigma–Aldrich (Saint-Quentin-Fallavier, France). Stock solutions were prepared in dimethylsulfoxide.

### Analysis and Statistics

Manual patch clamp results were analyzed with pCLAMP, version 9, software (Axon Instruments). APC results were analyzed with Patch MasterPro software (HEKA) for Patchliner^®^experiments and with Sophion Analyzer Software (Sophion Bioscience) for Qpatch^®^experiments.

Results are expressed as means ± SE of *n* observations. Data were compared using the Student’s *t*-test. Differences were considered statistically significant at *p* < 0.05. All statistical tests were performed using GraphPad Prism, version 6.0 (GraphPad Software).

## Results

### Evaluation of APC Whole-cell Recording Conditions

Automated planar patch clamp is commonly used to detect off-target effects on the potassium channel hERG during the safety testing phase of drug development. Here, we describe its use for recording currents carried by wt-CFTR and rescued F508del-CFTR channels and compare the results with those obtained from MPC recordings. In this study, we tested two APC devices: the Patchliner^®^from Nanion and the Qpatch^®^from Sophion Bioscience.

Both systems tested use the general principle of planar patch clamp, which is comparable to MPC with some differences in the experimental conditions (Milligan et al., 2009). Briefly, planar APC experiments use cartridge chips with a variable number of microfluidic chambers. Each chamber consists of two compartments containing extra- and intrasolutions, separated by a planar substrate (borosillicate glass for the Patchliner^®^, silicon-based glass for the Qpatch^®^) that has a micron-sized aperture. Cells in suspension are placed in the wells and interact with the glass to form stable giga-seal at the aperture. After giga-seal formation, whole-cell configuration is achieved by physical rupture of the cell-attached patch, then pre-programmed voltage-clamp steps are applied and recording begins. Both systems used the microfluidic technology but with the following differences: Patchliner^®^chips allow perfusion of external and internal solutions while Qpatch^®^has no internal perfusion. Another major difference is the higher throughput of the Qpatch^®^, which possesses one or two arms, each with four channels, while the Patchliner^®^has only a single pipetting arm with one channel (see for review, Dunlop et al., 2008).

As APC requires both fresh cell suspension and cell membrane integrity, the first step was to obtain viable and isolated cells in suspension. We tested the BHK, CHO, HeLa, and CFBE cell lines and several detachment protocols and reagents (e.g., Trypsin–EDTA, Accutase). The best quality cells in suspension were obtained using Accutase and the detachment protocol detailed above in the Section “Materials and Methods.” For all the tested cell lines, we achieved 90% cell viability (estimated by a manual counting of Trypan blue-stained cells) and well-separated cells without clusters.

Whole-cell-recording parameters and success rate for each cell line obtained with the Patchlinerx^®^system are shown in **Table 1** along with the number of experiments that reached our criteria for a successful recording: a seal resistance of 1 GΩ (*R*_m_), whole-cell access with a series resistance <20 MΩ (*R*_s_), and a complete recording sequence (i.e., basal conditions – CFTR activation – CFTR inhibition). Success rates varied between cell lines. The rates of achieving seals, entering the whole-cell configuration, and completing the CFTR recording protocol are shown in the table, with the overall success rates in pooled wt- and F508del-CFTR experiments of 31.1 and 25.6% in BHK and CHO cell lines, respectively. Much lower rates were obtained in CFBE epithelial cells (5.2%), although this might be improved with further optimization of the protocol. The following discussion focuses mainly on results obtained with BHK cells.

**Table 1:** Success rates of cystic fibrosis transmembrane conductance regulator (CFTR) recording with automated patch clamp (APC) experiments on Patchliner system.

Cell line	Number of channel	*R*_m_ > 1 GΩ	Whole cell (*R*_s_ < 20 MΩ)	Recording	%Success
Baby Hamster Kidney (BHK)	87	60	51	27	31.1
Chinese hamster ovary (CHO)	86	48	39	22	25.6
HeLa	133	71	57	18	13.5
Cystic Fibrosis Bronchial Epithelial (CFBE)	38	5	3	2	5.2

*Average recording parameters and success rates. Number of channel represents the number of experiment starting; *R*_*m*_ > 1 G*Ω*: number of cells reaching the giga seal; Whole-cell: number of whole-cell configuration obtained with a series resistance (*R*_*s*_) <20 M*Ω*; Recording: number of experiments with a complete recording sequence (basal – activation – inhibition).*

### Planar APC Recording of wt-CFTR

Recordings were collected and analyzed in experiments that met the above criteria. In the following experiments, voltage pulse protocols were applied under basal conditions, in presence of the activator cocktail containing 10 μM Forskolin (Fsk) + 30 μM Genistein (Gst) and after further addition of 10 μM CFTR_inh_172, a relatively selective CFTR inhibitor when added acutely (Ma et al., 2002). Briefly, Fsk regulates CFTR activity by stimulation of adenylate cyclase leading to the elevation of cAMP (Seamon and Daly, 1981) and PKA phosphorylation of the CFTR regulatory domain (R domain) (Tabcharani et al., 1991; Berger et al., 1993). The isoflavone Gst is a potent potentiator of the wt and F508del-CFTR (Hwang et al., 1997).

**FIGURE 2:**
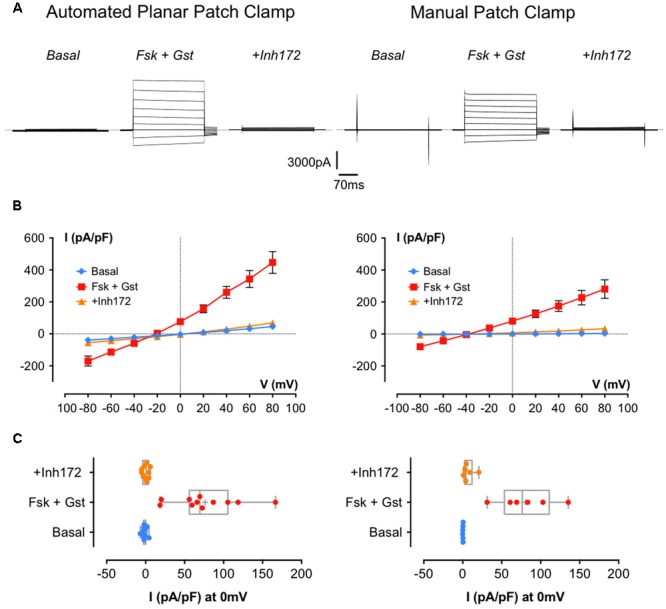
**Cystic fibrosis transmembrane conductance regulator whole-cell recording by automated and manual patch clamp from BHK-wt-CFTR. (A)** Representative traces of whole-cell Cl^-^ currents recorded by APC (left traces) or manual patch clamp (MPC; right traces) by stepping from a holding potential of -40 mV to a series of test potentials from -80 to +80 mV in 20 mV increments under basal (unstimulated) conditions or in the presence of 10 μM Fsk + 30 μM Gst or 10 μM CFTR_Inh_ 172 as indicated. *Dashed lines* indicate the zero current level. **(B)** Corresponding current densities (pA/pF) obtained by current–voltage relationships normalized by cell capacitance for APC (left traces; *n* = 11) and MPC (right traces; *n* = 6). **(C)** Corresponding distribution of current densities recorded at 0 mV. Error bars, SEM.

**Figure 2A** shows representative whole-cell currents recorded in BHK-wt-CFTR cells using both Patchliner^®^automatic (left traces) and conventional (right traces) methods. **Figure 2B** shows the corresponding *I/V* relationships. With the two experimental methods, Fsk + Gst elicited a robust Cl^-^ current that was almost abolished by the specific inhibitor CFTR_inh_172. However, the current density recorded by APC with Fsk + Gst was larger than the one recorded by MPC (respectively, at +60 mV 343.4 ± 52.8 pA/pF *n* = 11 and 227.4 ± 45.5 pA/pF *n* = 6; *p* = 0.16) and displayed slight rectification and a reversal potential shift to -20 mV from the theoretical E_Cl^-^_, which is expected to be near -40 mV. Several observations suggest that the differences observed between data obtained using APC and MPC were caused by a decrease in the seal resistance while recording.

First, there were no differences when we compared the cell capacitance (*C*_m_) and series resistance obtained with each method (*p* = 0.46 for *C*_m_ and *p* = 0.09 for *R*_s_; **Table 2**) suggesting the current density obtained with APC was not increased artificially due to underestimation of the cell capacitance or reduced access resistance to the inside of the cell. Comparing the current densities at +60 mV under control conditions revealed a significant difference between basal currents measured under APC and MPC conditions (32.3 ± 6.3 pA/pF *n* = 11 and 2.4 ± 0.4 pA/pF, *n* = 6, *p* = 0.003, respectively) and basal conductance was not sensitive to CFTR_inh_172 suggesting it was not mediated by CFTR (data not shown). By contrast, at 0 mV (where the non-selective leak current would be undetectable), current densities recorded in the presence of Fsk + Gst were similar for both methods (76.4 ± 12.9 pA/pF, *n* = 11 with APC and 80.5 ± 14.7, *n* = 6 with MPC; n.s. *p* = 0.85; **Figure 2C**).

**Table 2:** Comparison of patch clamp parameters for manual and automated BHK whole-cell recordings.

	Automated patch clamp (APC)		Manual patch clamp (MPC)	
	*C*_m_ (pF)	*R*_s_ (MΩ)	*C*_m_ (pF)	*R*_s_ (MΩ)
BHK-wt-CFTR	17.6 ± 5.0	18.9 ± 2.5	23.1 ± 2.8	12.4 ± 1.4
BHK-F508del-CFTR	16.4 ± 2.7	10.3 ± 1.3	17.9 ± 1.6	13.0 ± 1.4

*Mean of recorded parameters for complete experiment on BHK–CFTR (wt and F508del) obtained by APC (on Patchliner) or manual patch clamp (MPC); Cm: membrane capacitance; Rs: series resistance. Values are expressed as mean ± SEM.*

### APC Recording of Corrected F508del-CFTR

F508del is by far the most common CF mutation; therefore, the functional screening assay should enable the recording of F508del-CFTR currents. However, this mutation induces a gating defect in addition to the trafficking defect (Dalemans et al., 1991); therefore, the assay should be sensitive enough to accurately record potentiator-induced increases in Cl^-^ current so that different potentiator and corrector drugs can be compared. In another set of experiments, we compared F508del-CFTR current recordings obtained with the APC (Patchliner^®^) and MPC methods.

**FIGURE 3:**
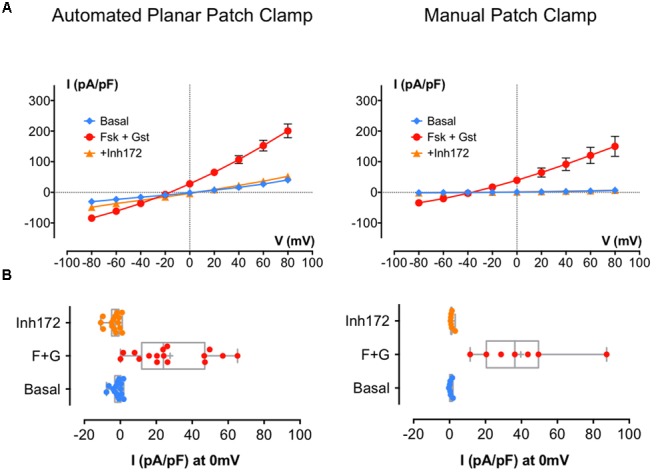
**Cystic fibrosis transmembrane conductance regulator whole-cell recording by automated and MPC from corrected (VX809 24 h) BHK-F508del-CFTR. (A)** Current densities (pA/pF) obtained by current–voltage relationships normalized by cell capacitance on BHK-F508del-CFTR corrected 24 h with 10 μM VX809. Whole-cell Cl^-^ currents are recorded by APC (left traces; *n* = 16) or MPC (right traces; *n* = 7) by stepping from holding potential of -40 mV to a series of test potentials from -80 to +80 mV in 20 mV increments under basal (unstimulated) conditions or in the presence of 10 μM Fsk + 30 μM Gst or 10 μM CFTR_Inh_ 172 as indicated. **(B)** Corresponding distribution of current densities recorded at 0 mV. Error bars, SEM.

First, we used BHK-F508del-CFTR cells under control conditions (DMSO treatment for 24 h). As expected, whole-cell recording detected little for any Cl^-^ current using either method (current densities at 0 mV: -0.67 ± 1.1 pA/pF, *n* = 10 with APC and 6.9 ± 2.9 pA/pF, *n* = 6 with MPC; data not shown). When the same experiments were performed after a 24-h pretreatment with the corrector VX809 (10 μM), CFTR current was detected. **Figure 3A** shows *I*/*V* relationships obtained using APC (left curves) and MPC (right curves) under basal conditions and during CFTR activation and inhibition. **Figure 3B** shows the corresponding distribution of each current density at 0 mV. As with wt-CFTR, Fsk + Gst elicited large F508del-CFTR currents that were nearly abolished by CFTR_inh_172 and, as previously observed, the APC current densities were somewhat larger and the reversal potential were shifted compared to MPC. Despite these differences, electrophysiological parameters were not significantly different (**Table 2**; *p* = 0.73 for *C*_m_ and *p* = 0.24 for *R*_s_) nor were the current densities at 0 mV (**Figure 3B**; 27.70 ± 4.9 pA/pF, *n* = 16 for APC and 39.62 ± 9.3 pA/pF, *n* = 7 for MPC, *p* = 0.23). Taken together, these results indicate that, aside from a slight contamination by leak current with APC, similar results can be obtained when recording rescued F508del-CFTR using either method.

### Variety of Recording Applications

**FIGURE 4:**
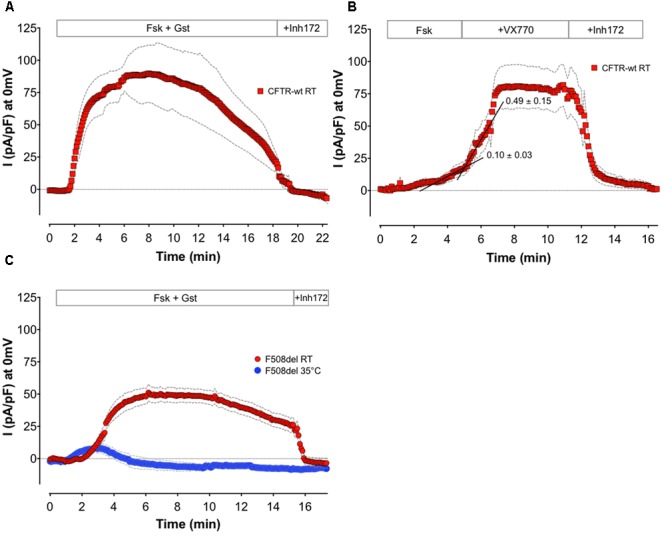
**Alternative application recorded with APC.** Means of whole-cell Cl^-^ current density time courses at 0 mV recorded on BHK-wt-CFTR **(A,B)** or BHK-F508del-CFTR **(C)** by APC. **(A)** Monitoring of wt channels rundown by long recording in presence of 10 μM Fsk + 30 μM Gst (*n* = 5). **(B)** Monitoring CFTR potentiation by sequential addition of 1 μM Fsk, and then 10 μM VX770 (*n* = 8). Black lines are representative linear regressions of the time course curves before and after potentiator addition. Means of slope values are indicated. **(C)** Effect of temperature on F508del channel function: recording of whole-cell current at room (RT; *n* = 6) or physiological temperature (35°C; *n* = 5) in presence of 10 μM Fsk + 30 μM Gst. For all conditions, 10 μM CFTR_inh_172 was added at the end of experiment. Colored symbols are the mean of current densities and gray dashed lines the corresponding error, SEM.

The recording sequence above is useful when determining the efficiency of candidate corrector molecules or comparing the potencies of several correctors. However, with MPC, we are potentially able to collect information such as the stability of currents over long time periods and the temperature dependence of channel gating. We examined the capabilities of APC for such experiments with Patchliner^®^system (**Figure 4**).

To study the stability of whole-cell currents, we performed APC whole-cell recordings on BHK cells over a 20-min period. **Figure 4A** shows the mean of wt-CFTR Cl^-^ current time courses in the presence of Fsk + Gst (*n* = 5). For these experiments, Rs were determined at the beginning and end of each recording and did not vary more than 25% from the initial value. As for MPC, we can record long enough with APC to observe the physiological rundown of CFTR channels (Becq et al., 1994). Such an experiment may be considered when assaying the ability of compounds to increase CFTR stability. In addition, the high quality of the seal and stability of the patch may allow the effects of sequential drug applications to be monitored and determination of EC_50_ or IC_50_. **Figure 4B** shows the mean of wt-CFTR current time courses at 0 mV with sequential addition of sub-maximal Fsk (1 μM) followed by 10 μM VX770 (*n* = 8). To evaluate the potentiator effect of the tested compound, linear regression analyses were performed using the points on each time course curve. For each individual experiment, points during the 2 min before and the 2 min after compound addition were used to calculate the slopes. The example in **Figure 4B** (black lines) shows representative linear regression before and after addition of VX770. Here, the mean slopes computed for eight different cells yielded values that are significantly different (0.101 ± 0.031 for Fsk alone and 0.499 ± 0.149 after 10 μM VX770 addition, *n* = 8, *p* = 0.02) and clearly demonstrate potentiation of the Fsk-activated current by the potentiator VX770.

Finally, the temperature dependence of F508del CFTR function was examined using APC. Whole-cell recordings were obtained at room temperature and at 35°C using BHK-F508del-CFTR cells that had been pretreated for 24 h with 10 μM VX809. **Figure 4C** shows the mean of current densities at 0 mV during stimulation of F508del-CFTR by Fsk + Gst at 22°C (*n* = 6) or 35°C (*n* = 5). According to the literature (Wang et al., 2011, 2014; Liu et al., 2012) and contrary to the CFTR response obtained at 22°C, only a small transient current is recorded at physiological temperature. These results show that whole-cell CFTR currents can be studied at 37°C using APC, allowing drug effects to be studied under physiological conditions. Also, it suggests the feasibility of screening for compounds that correct the thermal instability of F508del-CFTR.

### Planar APC Using Other Cells and Robotic Systems

**FIGURE 5:**
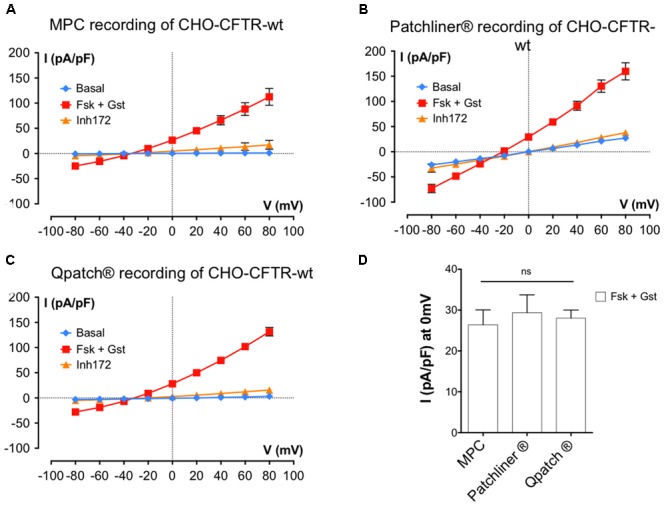
**Cystic fibrosis transmembrane conductance regulator whole-cell recording by automated and MPC from Chinese hamster ovary (CHO) cells stably expressing wt-CFTR. (A–C)** Current densities (pA/pF) obtained by current–voltage relationships normalized by cell capacitance on CHO-wt-CFTR. Whole-cell Cl^-^ currents are recorded with MPC (**A**, *n* = 4) or with APC with two workstations: the Nanion Patchliner (**B**, *n* = 10) or the Sophion Qpatch (**C**, *n* = 60) by stepping from a holding potential of -40 mV to a series of test potentials from -80 to +80 mV in 20 mV increments under basal (unstimulated) conditions or in the presence of 10 μM Fsk + 30 μM Gst or 10 μM CFTR_Inh_ 172 as indicated. **(D)** Histograms of current densities recorded at 0 mV in the presence of Fsk + Gst. Error bars, SEM; ns, not significant.

The experiments described above were performed using BHK cells and the Patchliner^®^APC system. However, as mentioned in **Table 1**, we also tested APC with different cell types. **Figures 5A,B** show *I*/*V* relationships obtained using MPC or APC to study CHO cells stably expressing wt-CFTR. Cells were recorded under basal conditions and in the presence of 10 μM Fsk + 30 μM Gst and 10 μM CFTR_Inh_ 172. With both methods, the activator cocktail Fsk + Gst, elicited strong voltage- and time-independent currents that were nearly abolished by CFTR_inh_172. A small shift in the reversal potential was observed with APC (-20 mV instead of the expected -40 mV); nevertheless, robust CFTR recordings were obtained with CHO cells stably expressing wt-CFTR, which resembled those obtained with BHK except for having lower current densities as expected with lower CFTR expression.

Finally, we tested CFTR recording using another robotic APC system: the Sophion QPatch^®^. Using QPatch^®^, the rates of success obtained were better for CHO-wt-CFTR cells, and the CFTR whole-cell recordings made with the same protocols and medium were close to those obtained previously (**Figure 5C**).

In summary, detailed comparison of the current densities at 0 mV using the MPC or two APC systems suggests they yield very similar results (**Figure 5D**; *I* (pA/pF) at 0 mV: 29.37 ± 4.4 pA/pF, *n* = 10 for the Patchliner^®^; 28.04 ± 1.9 pA/pF, *n* = 60 for Qpatch^®^ and 26.40 ± 3.6 pA/pF, *n* = 4 for MPC) and provide good reproducibility between different APC systems and cell models.

## Discussion

Several cell-based screening assays for CFTR are available that monitor anion flux [e.g., using radiotracer efflux (Venglarik et al., 1990) or YFP-based halide transport (Galietta et al., 2001)]. Although they provide high throughput, they are only semi-quantitative because the net electrochemical across the membrane for the anion changes during the course of the assay. Here, we have shown that planar APC can be used when screening for CFTR modulators and provides medium throughput together with several advantages over other cell-based assays.

APC allows wt- and corrected F508del-CFTR activity to be recorded with high efficiency and resolution approaching that of MPC, but at much faster rates. Optimizing the success rate is key to using these assays for drug testing. In the present study, success rates reached 31% for BHK and 25.6% for CHO cells, which are acceptable though values lower than that were found in the literature for similar cell lines and the same APC system (Milligan et al., 2009). To increase this rate of experiment success and develop the use of more physiological cell lines (i.e., CFBE and Calu-3), the cell preparation needs to be improved, e.g., by adapting protocols for culturing adherent cell lines in suspension (Muller et al., 2005).

CFTR recordings obtained using APC are similar to those obtained using MPC: no CFTR current was detected under basal conditions but it was strongly activated by Fsk + Gst and abolished by the specific CFTR inhibitor CFTR_inh_172. The main difference was that CFTR currents recorded using APC were often contaminated with non-selective leak currents due to a decline in seal quality. Since the user can’t easily intervene during the APC experiments, they require controls and well-designed protocols to allow the discrimination of false positives. In our experiments, we replaced potassium ions with cesium to block K^+^ currents and designed patch solutions with an E_Cl^-^_ far from 0 mV so that leak and CFTR currents could be easily discriminated.

Interestingly, APC can use the same protocols as MPC, making it useful for studies of current stability over long periods, the effects of sequential drug applications (useful for EC_50_ or IC_50_ determination, precise measurement of potentiation), and the temperature dependence of channel gating CFTR modulators. This last point is a real advantage compared to many HTS assay since it enables compounds to be tested at physiological temperature. As already mentioned, mutant CFTR channels have altered temperature sensitivity, yet, most screening assays are performed at room temperature, which may explain some discrepancies between *in vitro* results and clinical trials. Similar results were obtained using the two APC systems suggesting either planar APC methods can be used for CFTR studies.

Once the conditions have been optimized, APC is faster and easier to use than MPC with similar high-resolution results and the possibility to address the same questions. All steps during APC experiments are finely controlled and executed by a robot, allowing more consistent times and concentrations at which drugs are injected. The use of microfluidics within the APC chips ensures that volumes are minimized and allows more precise control of the temperature.

A major drawback with APC is the requirement for cells in suspension, which limits the use of epithelial cell lines under physiological conditions, including CFBE, Calu-3, and the human colonic cell line T84. The normal state of these cells is highly polarized, with tight junctions and apical CFTR. The effects of CFTR modulators may be influenced by the integrity of the epithelium and the maintenance of cell–cell contacts (Fritsch and Edelman, 1996). The detachment protocol used in this study or the generation of cell lines adapted to suspension, may also adversely affect interactions between CFTR and its partners, altering channel function and regulation.

## Conclusion

We have shown that whole-cell parallel planar APC is suitable for recording CFTR activity and gives robust results similar to those obtained by MPC but with higher throughput and standardized protocols. Screening with APC is an attractive option for pharmacological investigations including hit validation, trafficking correction, potentiation, thermal stability restoration, and the functional characterization of mutant CFTR.

## Author Contributions

AB, LF, JH, and FB designed the experiments. AB and LF performed experiments. AB and LF analyzed the data. AB, LF, JH, and FB wrote the manuscript.

## Conflict of Interest Statement

The authors declare that the research was conducted in the absence of any commercial or financial relationships that could be construed as a potential conflict of interest.
